# Complete Genome Sequences of *Salmonella enterica* Serovars Anatum and Anatum var. 15+, Isolated from Retail Ground Turkey

**DOI:** 10.1128/genomeA.01619-15

**Published:** 2016-01-21

**Authors:** Daya Marasini, Usama H. Abo-Shama, Mohamed K. Fakhr

**Affiliations:** a Department of Biological Science, The University of Tulsa, Tulsa, Oklahoma, USA; b Microbiology and Immunology Department, Faculty of Veterinary Medicine, Sohag University, Sohag, Egypt

## Abstract

The complete genome sequences of two isolates of *Salmonella enterica* serovars Anatum and Anatum var. 15+ revealed the presence of two plasmids of 112 kb and 3 kb in size in each. The chromosome of *Salmonella* Anatum (4.83 Mb) was slightly smaller than that of *Salmonella* Anatum var. 15+ (4.88 Mb).

## GENOME ANNOUNCEMENT

*Salmonella enterica* subsp. *enterica* serovar Anatum is one of the *Salmonella* serovars responsible for causing foodborne illnesses. *Salmonella* Anatum was responsible for causing a foodborne outbreak in association with enterotoxigenic *Escherichia coli* in Denmark in 2006 that was linked to pasta salad consumption (1). *Salmonella* Anatum-contaminated haylage in combination with the consumption of spoiled soybean meal was reported to cause bovine salmonellosis in lactating cows and calves (2). An outbreak of *Salmonella* Anatum occurred after consuming unpasteurized orange juice produced by a manufacturer in Florida in 1999 (3). *Salmonella* Anatum was also associated with the outbreak caused by the consumption of dried formula milk in 1996/1997 in France (4).

To date, and to our knowledge, there are only four *Salmonella* Anatum complete genomes available in GenBank other than the two reported in this study, and none of them have plasmids. We announce here the complete genome sequence of *Salmonella* Anatum strain GT-38 and *Salmonella* Anatum var. 15+ strain GT-01 isolated from ground turkey, which shows the presence of two plasmids in each strain.

Genomic DNA was isolated from overnight tryptic soya broth using the Qiagen DNeasy blood and tissue kit (Qiagen, Inc., Valencia, CA). The sample library for next-generation sequencing was prepared using the Nextera XT sample preparation kit and sequenced using an Illumina MiSeq (Illumina, Inc., San Diego, CA) with 121× coverage by the MiSeq version 2 reagent kit with 2 × 150 cycles. The sequence assembly was preformed using CLC Genomics Workbench version 7.5.1 and its Microbial Genome Finishing Module version 1.4 (Qiagen, Inc.). Automatic annotation was done using the NCBI Prokaryotic Genome Annotation Pipeline.

The complete genome of *Salmonella* Anatum strain GT-38 contained one chromosome of 4,830,607 bp in size, along with one larger plasmid (pDM04) of 112,176 bp in size and a smaller plasmid (pDM05) of 3,371 bp in size. The complete genome contained a total of 4,795 genes, of which 113 genes were present in the larger plasmid, and 4 genes were present in the smaller plasmid. The complete genome of *Salmonella* Anatum var. 15+ strain GT-01 contained a slightly larger chromosome of 4,879,815 bp in size, along with a larger plasmid (pDM02) of 112,176 bp in size and a smaller plasmid (pDM03) of 3,371 bp in size. The genome contained a total of 4,854 genes. The sequences of both the larger and the smaller plasmids were identical in the two strains.

Both of the isolates share high similarity to the *S. enterica* subsp. *enterica* serovar Anatum strain ATCC BAA-1592, which has a genome size of around 4.7 Mb. The larger plasmids were homologous to the previously sequenced plasmid pSH 148_107 of *S. enterica* subsp. *enterica* serovar Heidelberg, and the smaller plasmids were similar to that of *Salmonella* Heidelberg strain N13-01290-3-2. The larger plasmids contain genes coding for anti-restriction protein, β-lactamase, and ethidium bromide resistance protein, whereas the smaller plasmids contained mobility protein genes.

### Nucleotide sequence accession numbers. 

The chromosome, larger plasmid, and smaller plasmid sequences were deposited in GenBank under accession numbers CP013226, CP013224, and CP013225, respectively, for *Salmonella* Anatum strain GT-38, and under accession numbers CP013222, CP013221, and CP013223, respectively, for *Salmonella* Anatum var. 15+ strain GT-01.
